# Supplementary material for: Semi-rational evolution of a recombinant DNA polymerase for modified nucleotide incorporation efficiency
Source: PLoS One. 2025 Feb 14;20(2):e0316531. doi: 10.1371/journal.pone.0316531 (PMC11828419; doi:10.1371/journal.pone.0316531)
Supplement: S1 Raw images — (PDF) [file pone.0316531.s002.pdf]

S1_raw_images (corresponding to Supplementary Fig 1A)

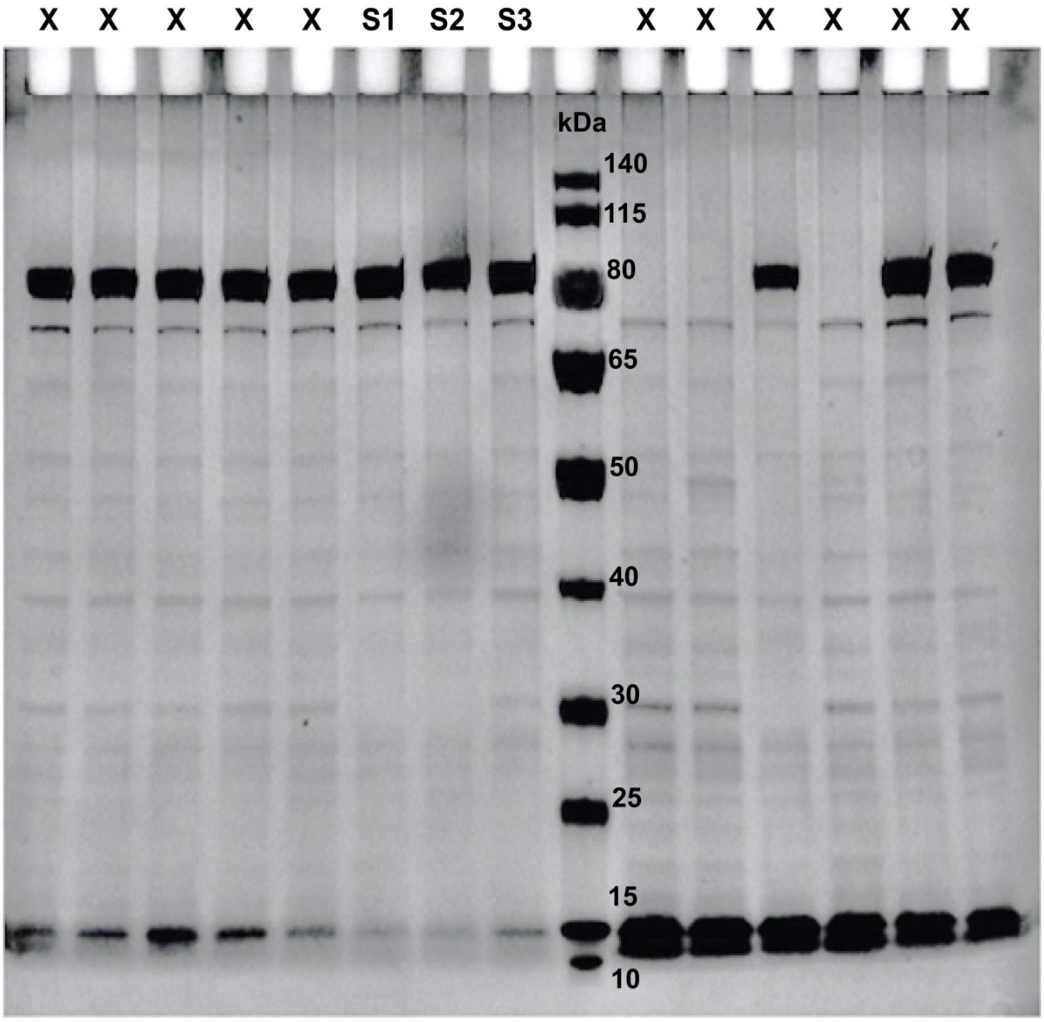

This is the raw figure for Supplementary Fig 1A that was generated from S1, S2, S3 panels.

The loading order of samples is from left to right. The gel image was captured using a Bio-Rad GelDoc XR imaging system at room temperature. This figure displays the SDS-PAGE analysis (12% gel) with WT KOD pol represented by S2, KOD variant Mut_1 represented by S1, and KOD variant Mut_C2 represented by S3. The 10 µL volume of samples loaded was around 1 µg, and the purification method involved lysing cells with lysozyme at 37 °C for 10 min and centrifugation after heating at 80°C for 30 min. The gel was run at 120V and 1-2 h at room temperature. (Details in Materials and Methods section)

S2_raw_images (corresponding to Supplementary Fig 4)

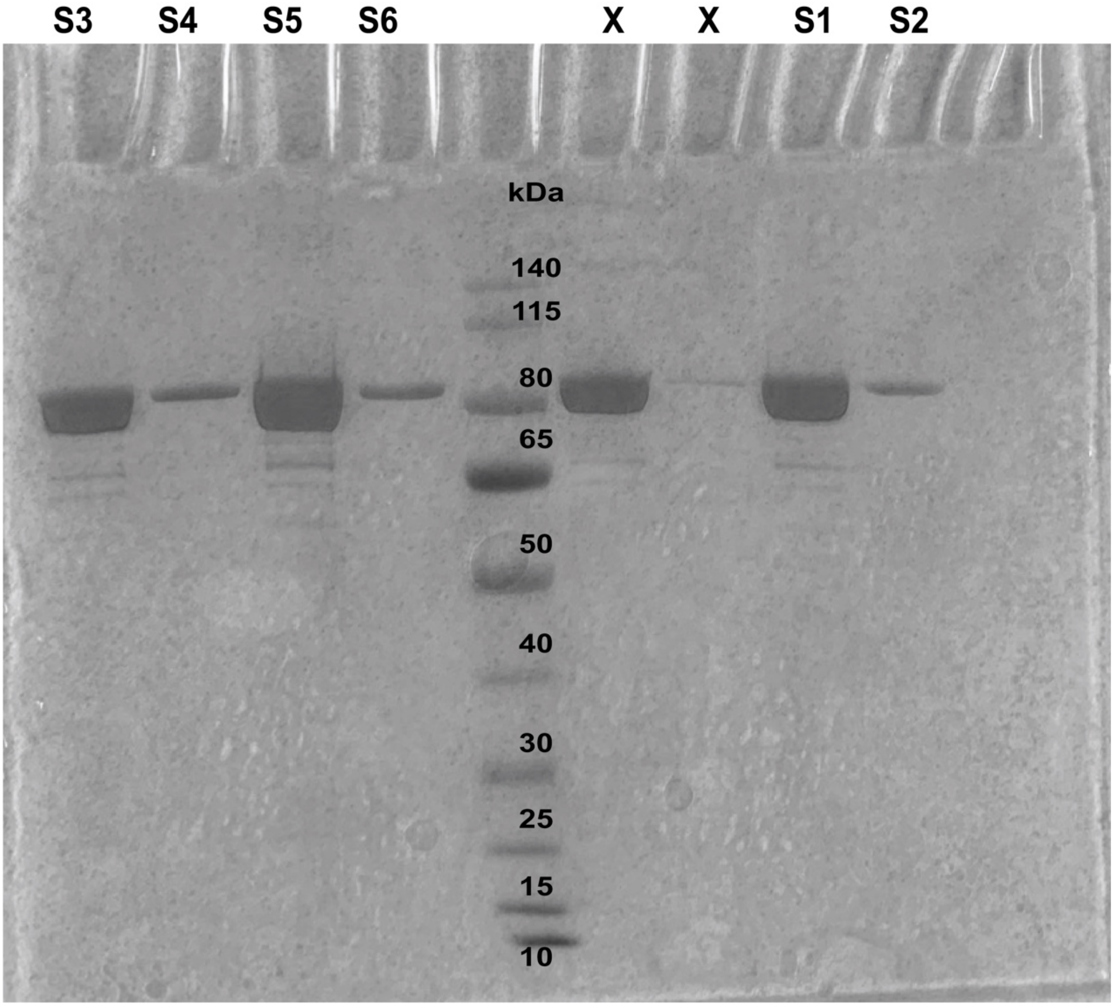

This is the raw figure for Supplementary Fig 4 that was generated from S1~S6 panels.

The loading order of samples is from left to right. The gel image was captured using a Bio-Rad GelDoc XR imaging system at room temperature. The SDS-PAGE analysis (12% gel) for rigorously purified KOD mutant proteins. WT KOD pol represented by S1 (5 µg) and S2 (0.25 µg), KOD variant Mut_C2 represented by S2 (5 µg) and S4 (0.25 µg), and KOD variant Mut_E10 represented by S5 (5 µg) and S6 (0.25 µg). We employed a three-step purification process involving Ni affinity chromatography and anion-cation exchange chromatography using ÄKTA. The estimated protein purity was approximately 95% based on ImageJ analysis of the gels. (Details in Materials and Methods section)
